# Heterogenous effect of early adulthood stress on cognitive aging and synaptic function in the dentate gyrus

**DOI:** 10.3389/fnmol.2024.1344141

**Published:** 2024-04-04

**Authors:** Eun Hye Park, Yong Sang Jo, Eun Joo Kim, Eui Ho Park, Kea Joo Lee, Im Joo Rhyu, Hyun Taek Kim, June-Seek Choi

**Affiliations:** ^1^School of Psychology, Korea University, Seoul, Republic of Korea; ^2^Department of Psychology, New York University, New York, NY, United States; ^3^Department of Psychology, University of Washington, Seattle, WA, United States; ^4^Department of Anatomy, Korea University College of Medicine, Seoul, Republic of Korea; ^5^Department of Structure and Function of Neural Network, Korea Brain Research Institute, Daegu, Republic of Korea

**Keywords:** cognitive aging, stress, memory, dentate gyrus, synaptic density

## Abstract

Cognitive aging widely varies among individuals due to different stress experiences throughout the lifespan and vulnerability of neurocognitive mechanisms. To understand the heterogeneity of cognitive aging, we investigated the effect of early adulthood stress (EAS) on three different hippocampus-dependent memory tasks: the novel object recognition test (assessing recognition memory: RM), the paired association test (assessing episodic-like memory: EM), and trace fear conditioning (assessing trace memory: TM). Two-month-old rats were exposed to chronic mild stress for 6 weeks and underwent behavioral testing either 2 weeks or 20 months later. The results show that stress and aging impaired different types of memory tasks to varying degrees. RM is affected by combined effect of stress and aging. EM became less precise in EAS animals. TM, especially the contextual memory, showed impairment in aging although EAS attenuated the aging effect, perhaps due to its engagement in emotional memory systems. To further explore the neural underpinnings of these multi-faceted effects, we measured long-term potentiation (LTP), neural density, and synaptic density in the dentate gyrus (DG). Both stress and aging reduced LTP. Additionally, the synaptic density per neuron showed a further reduction in the stress aged group. In summary, EAS modulates different forms of memory functions perhaps due to their substantial or partial dependence on the functional integrity of the hippocampus. The current results suggest that lasting alterations in hippocampal circuits following EAS could potentially generate remote effects on individual variability in cognitive aging, as demonstrated by performance in multiple types of memory.

## 1 Introduction

One of the hallmark features of cognitive aging is its heterogeneity, both among and within individuals. Some individuals maintain remarkable cognitive skills until final stages of life, while others show severe declines with age (inter-individual variability). Additionally, within the same elderly individual, some forms of cognitive functions are preserved relatively better than others (intra-individual variability). Aging-related variability in cognitive capacity has been observed in rodents, non-human primates and humans (Barnes, 1987; Barnes, 1988; Erickson and Barnes, 2003; Burke et al., 2012; Engle and Barnes, 2012; Leal and Yassa, 2015), underscoring its complex nature resulting from the interplay between genetic and environmental factors (Hultsch et al., 2008; Gillespie et al., 2022).

As a prevailing external factor, stress has both immediate and long-lasting effects on cognitive functions (Kim and Diamond, 2002; Brunson et al., 2005; Champagne et al., 2008; Sandi, 2013). Previous studies indicate that stress experience during the postnatal period (Brunson et al., 2005; Kosten et al., 2006, 2007) and the mid-adulthood period (Sandi and Touyarot, 2006; Borcel et al., 2008) can lead to cognitive impairment later in aged animals. Stress during early adulthood is often underestimated despite being a critical phase in life. During this period, the nervous system, including cortical structures, undergoes significant maturation, while individuals face new behavioral challenges as they navigate independent life patterns (Gogtay et al., 2004; Matud et al., 2020). These challenges, coupled with limited experience in handling various life threats in nature, subjects individuals to stress, which can profoundly affect the brain circuits involved in cognitive processes (McEwen, 1999, 2011; Lupien et al., 2018). Despite the importance of early adulthood stress (EAS) on the cognitive functions in subsequent life stages, few prior studies have examined multiple forms of cognitive functions following EAS using animal models.

The hippocampal-entorhinal cortical areas are crucial in memory and cognitive functions (Leal and Yassa, 2015; Geiller et al., 2017; Marks et al., 2021; Zhang et al., 2023). In this circuit, the DG serves as a major gateway into the hippocampus, receiving inputs from the entorhinal cortex through the perforant path and controlling information processing to CA3-CA1 (Witter, 2007; Drew et al., 2013; Dvorak et al., 2021). The functional role of the DG is a neural computing process that discriminates between similar memories through the regulation of local inhibitory and excitatory machinery (Marr, 1971; Leutgeb et al., 2007; Neunuebel and Knierim, 2014; Park et al., 2015; van Dijk and Fenton, 2018). In aged rats, the cortical inputs to the dentate gyrus were substantially reduced compared to the subsequent intrinsic connection of CA3 and its outputs to CA1 (Geinisman et al., 1992; Smith et al., 2000; Geinisman et al., 2004). Long-term stress exposure leads to a continuous suppression of neurogenesis and modification in the structure of the dentate gyrus (Alves et al., 2018; Chenani et al., 2022; Segi-Nishida and Suzuki, 2022; Sun et al., 2023). Therefore, the DG might be a critical area for comprehending cognitive function changes in the aging process with exposure to early adulthood stress.

In this study, we employed chronic mild stress (CMS) to examine the long-lasting effect of EAS on cognitive functions later in life. Specifically, we focused on the declarative memory functions including recognition memory (RM) using the object recognition test, episodic-like memory (EM) using the paired-associate test, and trace fear memory (TM) using trace fear conditioning. To probe the neural underpinnings of the EAS effect on cognitive aging, we also examined the physiological and anatomical characteristics in DG with particular focus on synaptic plasticity, cell density and synaptic density.

## 2 Materials and methods

**Figure 1:**
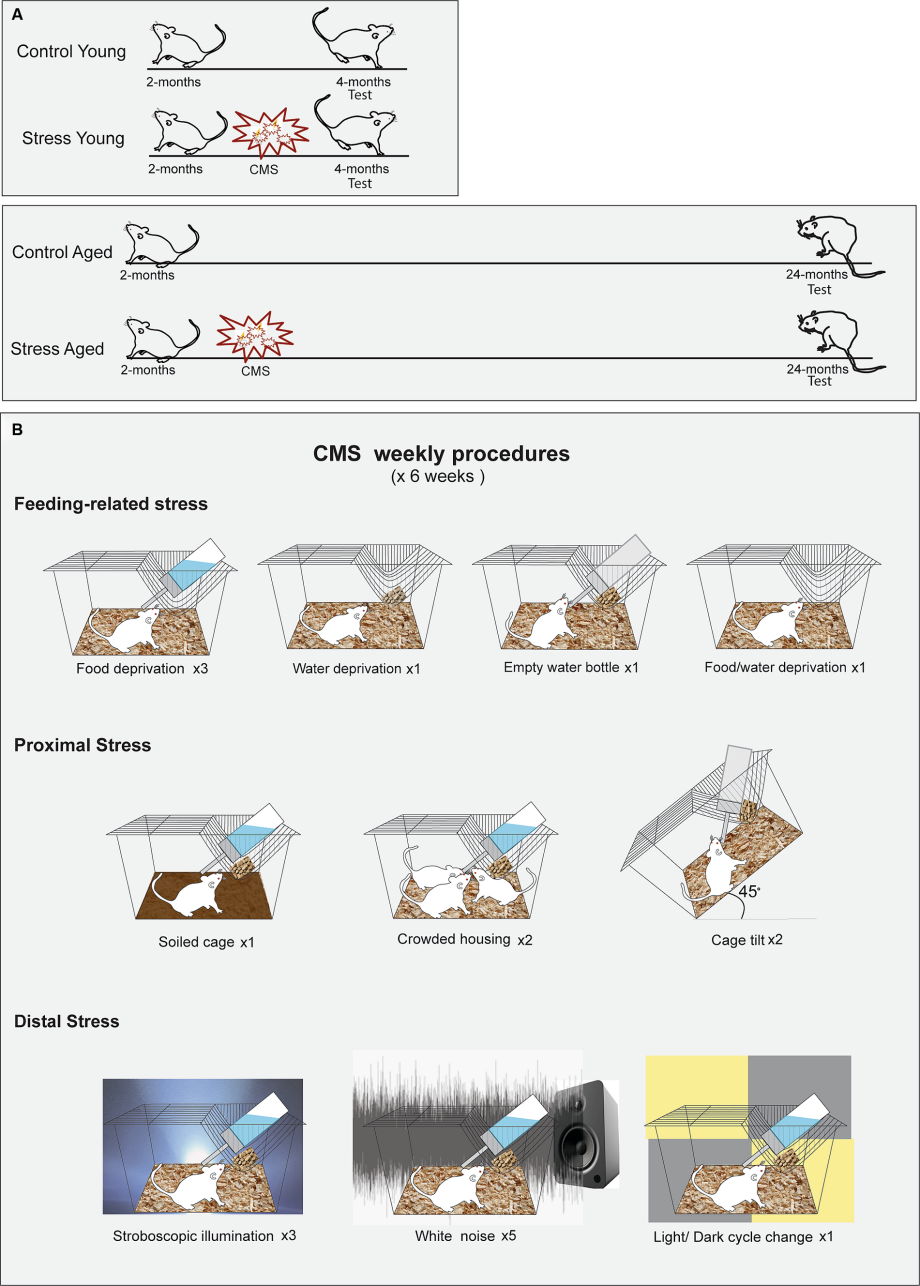
Experimental protocols for the chronic mild stress (CMS). **(A)** Timing of CMS for the young and aged rats. Rats in both age groups experienced a scheduled stress treatment for six weeks, beginning at 2 months of age. **(B)** Illustrated schedule for CMS. The rats were subjected to ten different manipulations, chosen from one of the three stress categories, following a predetermined sequence.

Adult male Wistar rats (SLC Inc., Hamamatsu, Shizuoka, Japan) were assigned to four groups based on age and stress treatments (CY: Control Young, *n* = 15, SY: Stress Young, *n* = 15; CA: Control Aged, *n* = 8, SA: Stress Aged, *n* = 12). Stress was administered to the stress groups (SY and SA) at the age of two months. The memory tests were administered at different time points: Two weeks after the last day of the stress procedures for the young groups (CY and SY) and 22–24 months for the aged groups (CA and SA) (Figure 1). The stress groups were exposed to six weeks of chronic mild stress (CMS), including food deprivation, water deprivation, empty water bottle, soiled cage (300 mL of water/l of sawdust bedding), crowded housing, cage tilt (45°), stroboscopic illumination, intermittent white noise (70 dB), and light/dark change (Papp et al., 1996; Willner et al., 1996). These stressors were intermixed for a 1-week schedule (Supplementary Table S1), which was repeated for six weeks. The control rats were housed in a separate room and received regular care without any of the aforementioned stressors. All animals were kept on a 12-h reversed light/dark cycle with access to food and water *ad libitum*. All behavioral tests were given during the dark phase.

### 2.1 Behavioral tests

#### 2.1.1 The object recognition test

**Figure 2:**
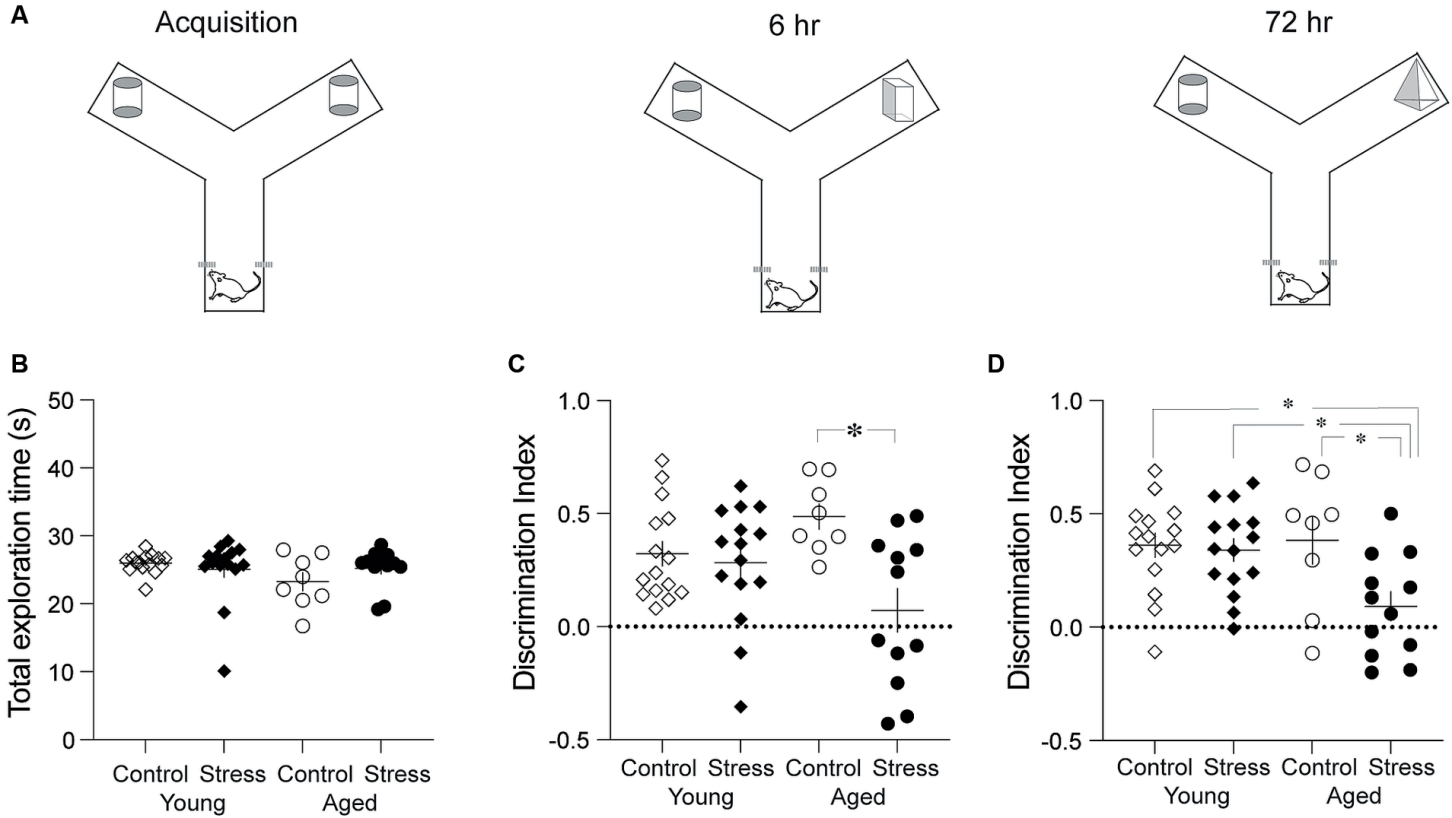
Object recognition memory test. **(A)** Schematic diagram showing three phases. The three phases include Acquisition, 6-h and 72-h Retention tests. **(B)** Results from the acquisition phase. No differences were observed among all groups in the total time spent exploring the objects. **(C)** Results from the retention test conducted 6 h after the acquisition. The Stress Aged group exhibited significantly lower discrimination between two objects compared to the Control Aged group. **(D)** Results from the recognition memory test conducted 72 h after the acquisition. The Stress Aged group exhibited significantly lower discrimination between two objects compared to the other three groups (*p < 0.05 for all figures).

To assess recognition memory (RM), the novel object recognition test was performed using a modified Y-maze. Each arm of the Y-maze was 30 cm in length, 15 cm in width, and 30 cm in height (Figure 2A). A video camera was mounted above the maze to record the behavior. The test was conducted in three phases: habituation, acquisition, and retention (Figure 2A). On day 1, the rats were habituated to the maze by freely exploring for 5 min. On day 2, the rats were placed in the starting arm and released into the maze with two identical objects at the end of the arms (acquisition) (Figure 2B). Object exploration was defined as directing the nose within 1 cm from each object. The acquisition phase ended when the rat had explored either object (two identical objects, designated as A or A’) for a total of 20 s or had been in the maze for 10 min, whichever occurred first. The retention test was given 6- and 72 -h after the acquisition phase. In the 6-h retention test, the rat was reintroduced into the Y-maze where one of the familiar objects was replaced by a novel one (A and B). The duration of time spent exploring each object was recorded for 3 min. In the 72-h retention test, the novel object (B) in the previous session was replaced by yet another novel object (C). The rat was allowed to explore the objects for 3 min. All three objects were counterbalanced across animals. The object discrimination index was calculated by the following equation (T_N_ – T_F_)/(T_N_ + T_F_), in which T_N_ and T_F_ represent time spent exploring the novel object and the familiar object, respectively.

#### 2.1.2 The paired-association test

**Figure 3:**
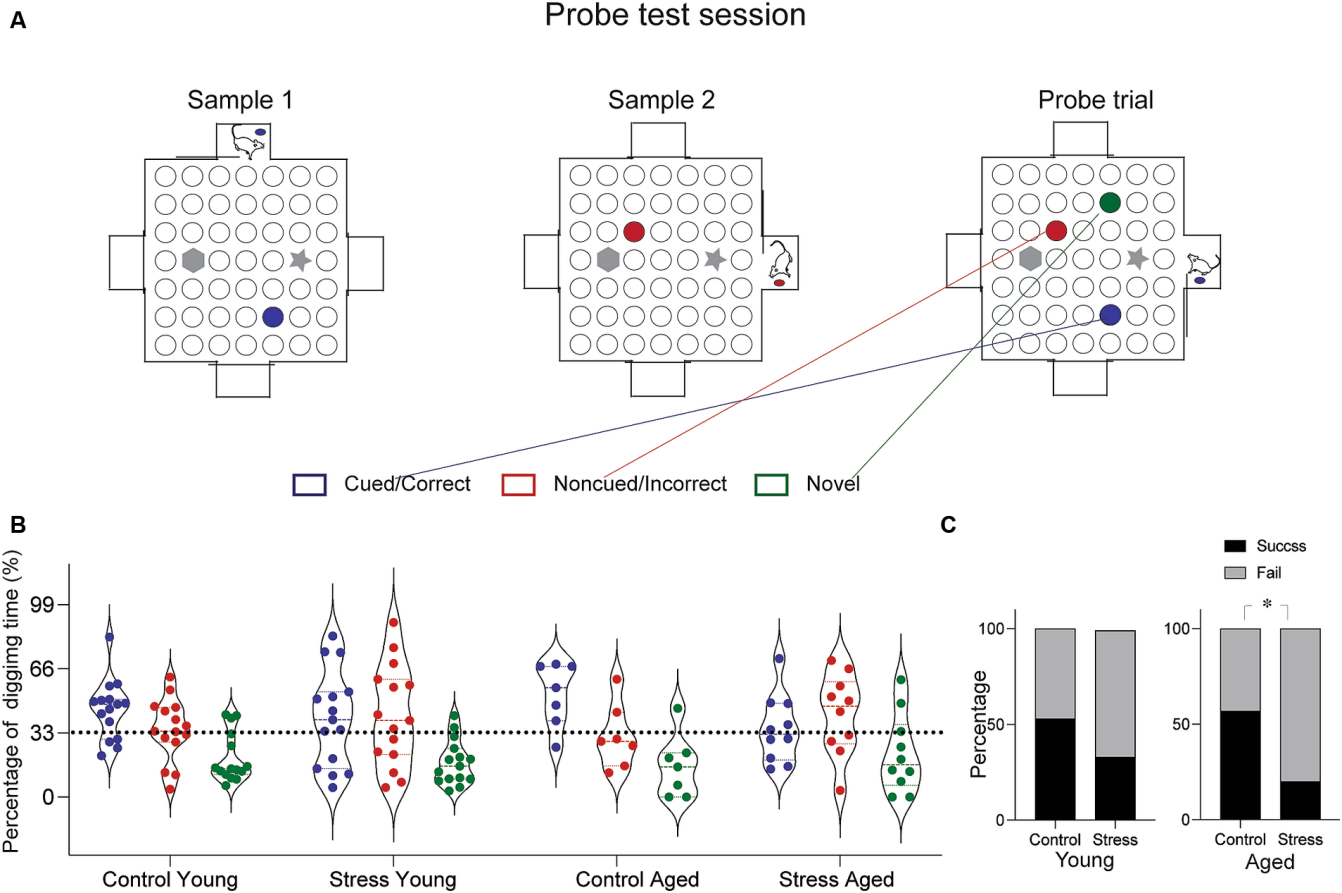
Episodic-like memory test. **(A)** Schematic diagram of the test arena and experimental protocol for the probe test. In this session, the rats were given a chance to learn two sets of flavor–place associations (Sample 1 and Sample 2, marked by blue and red circles). Following the sample trials, a probe test trial presented three different sand well locations open—the cued, the non-cued and the novel distractor (marked blue, red and green, respectively). Rats were provided a pellet matching the cued location before entering the arena and were allowed to explore freely for 90 s. Time spent digging at each well was measured. **(B)** Results from the probe trial. Percentage of digging time at each sand well was compared across different groups. **(C)** Percentage of rats showing successful retrieval. The percentage of subjects in each group demonstrating successful retrieval was assessed based on the proportion of digging (Cued > Noncued > Novel well in the order of digging time, was considered a success, and fail otherwise). The Stress Aged group was less likely to perform successful retrieval than the Control Aged [*X*^2^ 1, (*N* = 17) = 5.61, *p < 0.05].

To evaluated episodic-like memory, the rats were trained and tested on a paired-association task (Day et al., 2003; Bast et al., 2005). The arena was made of white particle board with a melamine surface (1.4 m × 1.4 m) surrounded by side walls made of clear Plexiglas (30 cm). Four start boxes (30 cm × 30 cm) were centered on each of the four walls with a sliding door leading into the arena. The arena surface contained forty-nine small sand wells (7 cm in diameter, 4 cm in depth), evenly spaced and covered with particle board lids. Out of 49 locations, two were chosen to have visually distinct landmarks. Instead of sand well, two tall objects (20 cm in height) were place at these specific locations (Figure 3A, gray marked position). During training, rats were given restricted food access and maintained 85% of their body weight throughout the experiment. The habituation period lasted five days, during which the rats were trained to dig through the sand wells to retrieve regular food pellets. Initially, the food pellets were placed on the surface of the sand and gradually deeper into the well as training progressed. After the habituation phase, the rats were trained in the paired-association task, that is distinct flavors of food pellet were associated with specific location within the arena. A total of twenty-two flavors were used throughout all the experiment (Supplementary Table S2). Each combination of flavor and location was used only once per training session to avoid any potential confusion. Each training session consisted of two sample trials and a choice trial. In a sample trial, the rats were introduced to a specific flavor inside the start box first and then allowed to approach the well containing the same flavor, where they would dig to find the buried food pallet. This procedure was repeated for another flavor (Supplementary Figure S1, Yellow and Green marked location for each sample trial). In a choice trial, the rats were presented with a sample cue flavor in the start box and given the opportunity to choose one of the two sample wells. A reward was buried only in the well matching the cue flavor. The latency to arrive at the correct well location during the choice trial was measured in daily training. After ten days of paired-association training, rats underwent to the probe test session for episodic-like memory, comprising two sample trials and one probe trial. The probe trial differed from choice trial (training day) in two ways: three wells were opened (the two from the sample trials and one as a distractor), and none of the wells contained a reward (Figure 3A). Digging times were measured during 90 s cut-offs.

#### 2.1.3 Trace-fear conditioning

**Figure 4:**
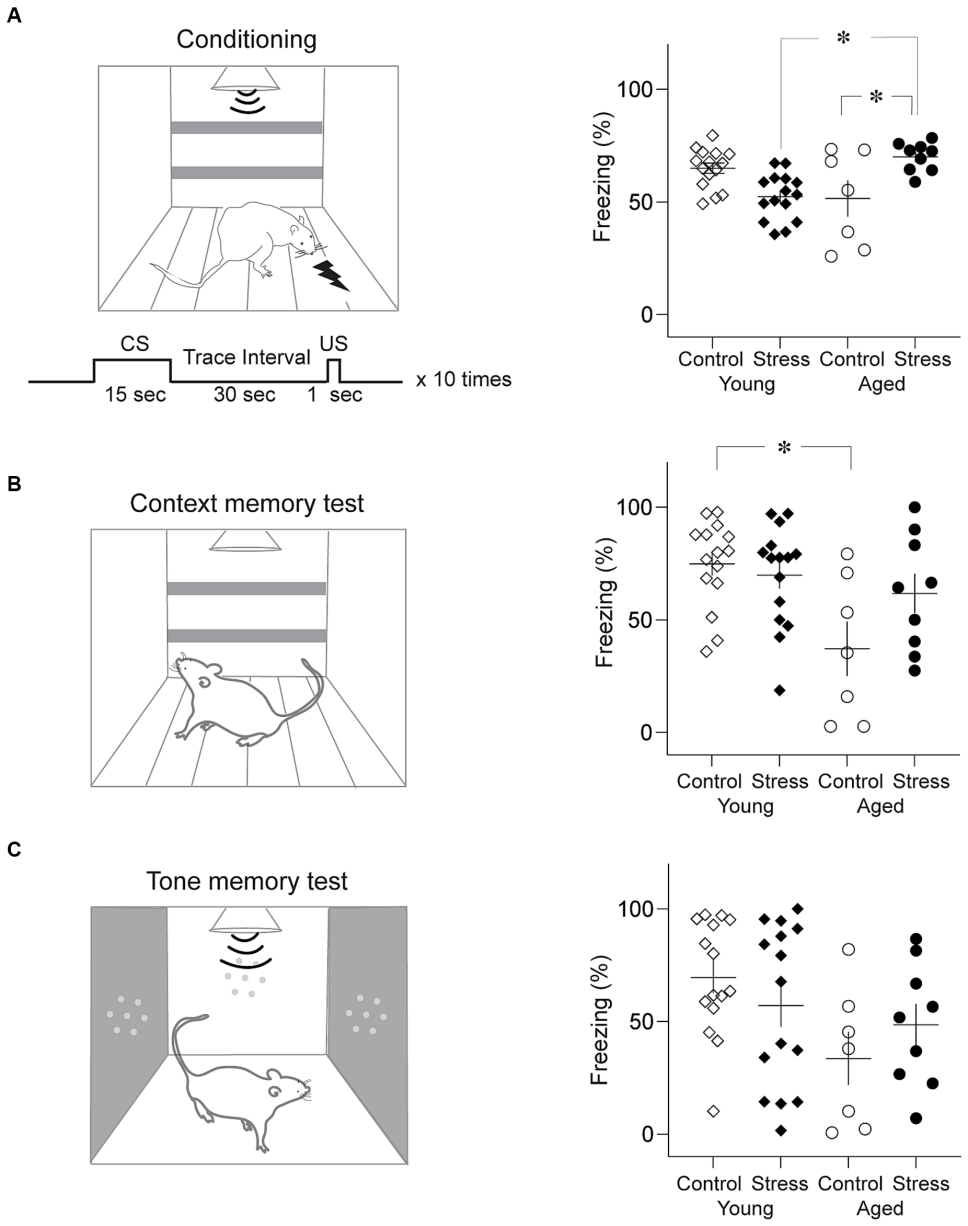
Trace fear memory test. **(A)** Results from trace fear conditioning. Schematic diagram of conditioning chamber and trace fear conditioning protocol (Left) and average freezing level to the tone CS (Right). Stress Young rats showed significantly less freezing than Control Young while Stress Aged rats froze more than Control Aged rats. **(B)** Results from the context memory test. Schematic diagram of context test chamber (Left) and average freezing level to the context (Right). Control Aged rats showed significantly less freezing than Young rats regardless of stress condition. **(C)** Results from the tone memory test. Schematic diagram of the test chamber (Left) and average freezing level to the tone CS (Right). No group difference was found (*p < 0.05, all figures).

Trace fear conditioning was conducted in a conditioning apparatus that has been used in our previous studies (Seo et al., 2008) as shown in Figure 4. The exterior dimensions of the conditioning chamber were 30 cm (L) × 25 cm (L) × 25 cm (H), with black Plexiglas walls and a metal grid floor. This conditioning chamber was placed in a sound-attenuated chamber equipped with a ventilating fan. A shock generator (E 13–14, Coulbourn Instruments, Allentown, PA, USA) was used to generate a scrambled foot shock. A camera was mounted on the ceiling to record the animal’s behavior. On day 1, rats were habituated to the training chamber and 10 tone CSs for 18 min. On day 2, the rats were placed in the conditioning chamber and subjected to the conditioning session (Figure 4A, left). After 2 min in the conditioning chamber, rats received ten trials of CS-US trace conditioning. A 15-s tone CS (4 kHz, 80 dB tone), 30-s trace interval, and 1-s foot shock US (1 mA) were delivered on each trial (300-s inter-trial-intervals). After conditioning, rats were transported to their home cage. Next day, rats were placed in the conditioning chamber for 5 min without the CS for contextual fear memory testing (Figure 4B, left). Four hours after the context test, rats underwent a tone fear memory test where a 5-min continuous tone (4 kHz, 80 dB) was presented following 2-min of habituation in a novel testing chamber (27 cm (L) x 25 cm (W) × 34 cm (H); an aluminum wall and no metal grid: Figure 4C, left). Freezing was scored off-line using the recorded video files by two observers blind to the identity of the rats. Freezing was defined as lack of movement for more than 1 s.

### 2.2 *In vivo* field potential recording in the dentate gyrus

**Figure 5:**
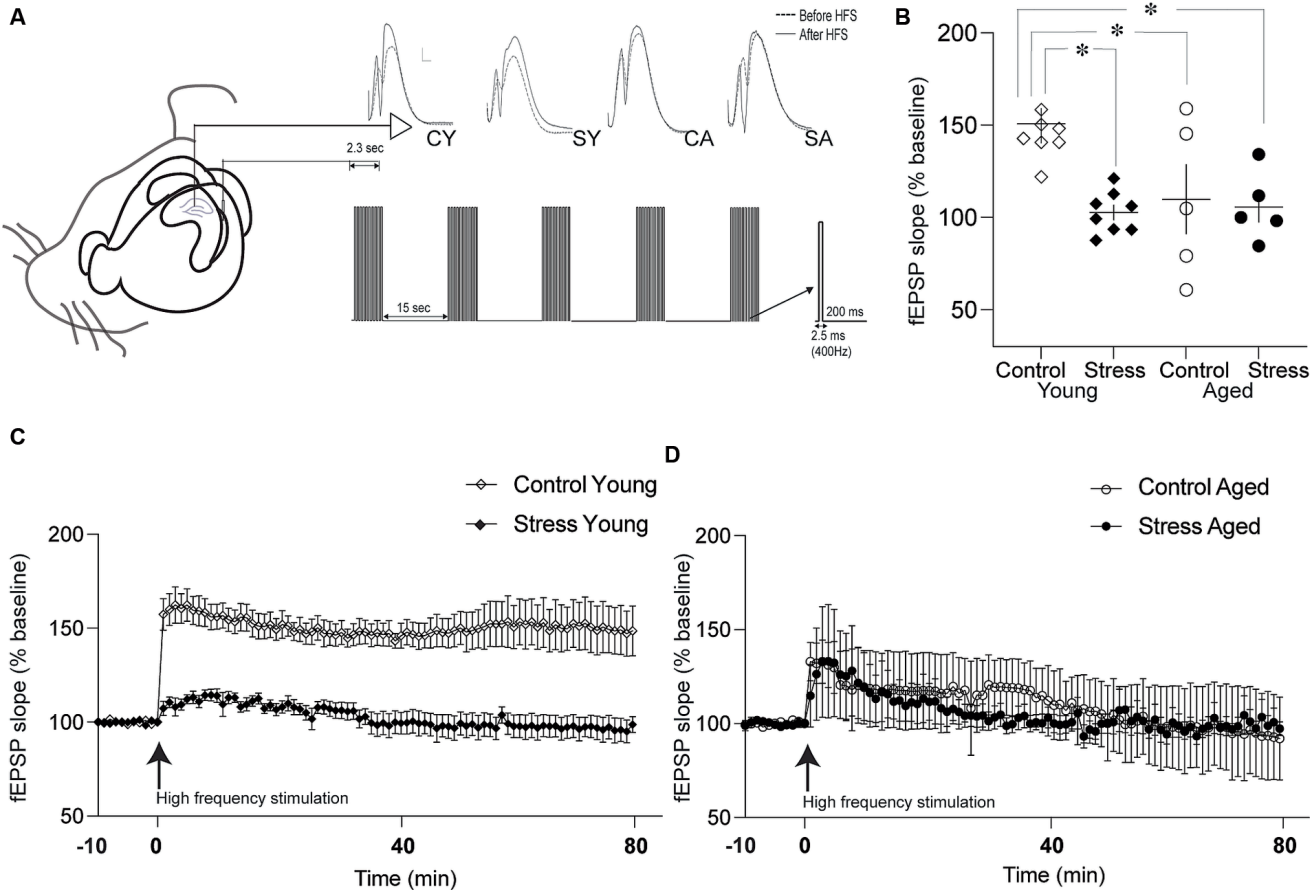
Synaptic plasticity in the dentate gyrus (DG). **(A)** Schematic drawing of recording and stimulation protocols for long-term potentiation (LTP) in the DG-perforant synapse. Representative evoked field potentials before (dotted line) and after (solid line) LTP induction were depicted on the top. CY, control young; SY, stress young; CA, control aged; SA, stress aged. **(B)** Changes in the fEPSP slope following LTP induction. The fEPSP slopes of Stress Young, Control Aged and Stress Aged groups were reduced compared to Control Young group. **(C)** Time course of LTP in young rats. The fEPSP was monitored for 80 min following high frequency stimulation to induce LTP. **(D)** Time course of LTP in aged rats. The LTP was induced following high frequency stimulation but degraded shortly (*p < 0.05).

After completing all behavioral test, a random subset of the rats from each experimental group underwent electrophysiological assessment to examine synaptic plasticity in the dentate gyrus (DG). The rats were anesthetized with sodium pentobarbital (100 mg/kg, i.p) and placed on a stereotaxic frame. A recording electrode (epoxy-coated tungsten electrode, 125 um in diameter, impedance 1–6 MΩ) and a bipolar stimulating electrode were ipsilaterally inserted in DG and perforant path (PP), respectively, with the following coordinates: AP: −3.8, ML:2.0 and DV: 3.5 to 4.9 for DG, AP: −7.8, ML: 4.0, DV −3.8 to −5.9 for PP. Baseline evoked potentials were recorded at every 15-s for 10 min. The stimulus intensity was determined to be 50% of the intensity (200–500 μA) that produced the maximum field excitatory postsynaptic potential (fEPSP) measured by the slope. To induce long-term potentiation (LTP), the tetanic high frequency stimulation is delivered to DG. This stimulation consisted of 5 trains, each comprising 10 pulses of 2.5 ms duration, with an inter- train interval of 15-s (Figure 5A). Following the tetanic stimulation, test stimulation was delivered at every 15-s for 80 min. The evoked potentials were amplified (× 1,000), filtered at 0.1 kHz −10 kHz band width, and digitized at 10 kHz. The fEPSP slope was computed off-line after all recordings were completed.

### 2.3 Electron microscopy

#### 2.3.1 Tissue preparation

A subset of the rats from each group was used for electron microscopic analysis. The rats were anesthetized with sodium pentobarbital (100 mg/kg, i.p), and perfused with the fixative (2% paraformaldehyde, 2.5 glutaraldehyde in 0.1 M phosphate buffer, pH 7.4). The brains were removed and sectioned coronally (100 μm thickness) using a vibratome. The tissue containing the hippocampus was dissected out. The tissues were post-fixed in 1% osmium tetroxide and stained *en bloc* in a 2% uranyl acetate solution. Samples were dehydrated through a series of alcohol washes, transferred into propylene oxide, and embedded in an Eponate12™ embedding kit (Ted Pella, Inc., CA, USA).

#### 2.3.2 Estimation of granule cell density

**Figure 6:**
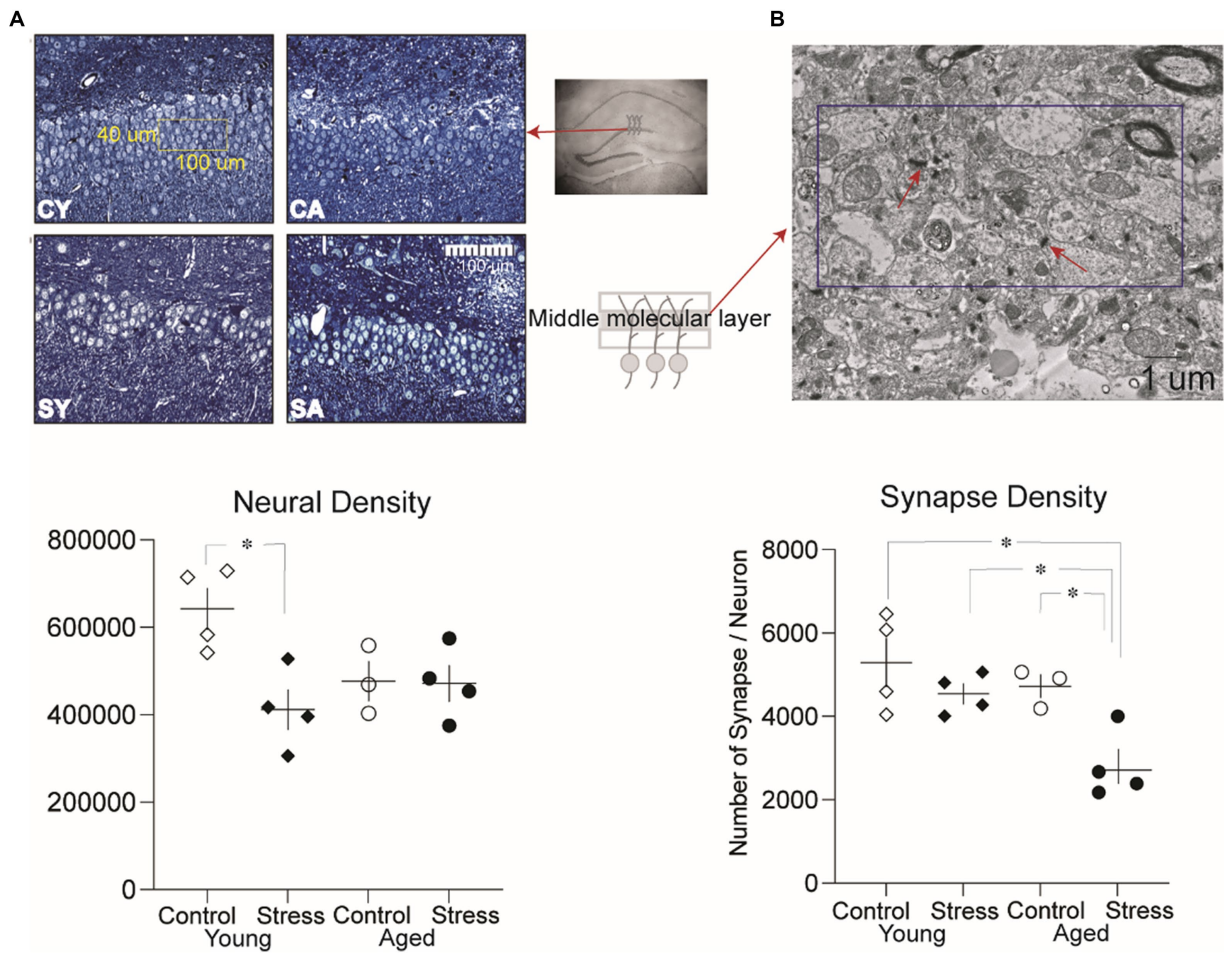
Analysis of neural density and synaptic density in the dentate gyrus. **(A)** Representative microscopic images of the granule cell layer from each group and neural density in the dentate gyrus. A significantly lower neural density was observed between the Stress Young group compared to Control Young. CY, control young; SY, stress young; CA, control aged; SA, stress aged. **(B)** Representative electron micrographic images of the middle molecular layer where the synaptic density was measured (dissector size:10 um × 5 um). A significantly lower synaptic density per neuron was observed in the Stress Aged group compared to all three other groups (*p < 0.05).

From the tissue blocks, serial semi-thin sections (1 μm in thickness) were collected using a microtome (Reicher-Jung Ultracut-E, Leica Microsystems, Wetzlar, Germany). After toluidine blue staining, digital images (40 ×) were acquired of the granule cell layer using a light microscope (Zeiss, Oberkochem, Germany). The serial micrographs were aligned using software program (Reconstruct^1^). The granule cell density was estimated based on the “Physical dissector” principle (Sterio, 1984; Gundersen et al., 1988) at the dorsal limb of dentate gyrus. Pairs of images were compared in succession. The first image was the ‘reference’ section and its pair the ‘look-up’ section. Dissector frames (100 μm × 40 μm) were randomly placed on the individual reference images (Figure 6A). If a cell sampled in ‘reference’ section was not seen in the ‘look up’ section, the cell was counted (Sterio, 1984, Gundersen et al., 1988). Granule cell density was calculated using the following formula: Nv = Total number of counted cells/Sum of Areas of disector frame × Section thickness (Sterio, 1984).

#### 2.3.3 Estimation of medial perforant path to granule cell synapses

To estimate the synaptic density, the tissue blocks were further trimmed down to the molecular layer of dentate gyrus. Serial thin sections (80 nm-thickness) were mounted on formvar-coated slot grids. The molecular layer was divided into three sections, and the middle section was chosen for further examination for synapses innervated by the medial perforant path (Witter, 2007; Gray and Barnes, 2015). Serial electron micrographs were taken at a magnification of 15,000 × using a Hitachi H-7500 transmission electron microscope (Hitachi, Tokyo, Japan). After aligning the images, the synaptic density was estimated using the physical dissector principle. For each animal, three to five sets of three serial images were analyzed. Synapses were identified based on the presence of synaptic vesicles and a clearly distinguishable postsynaptic density. If a synapse sampled in the reference sections (dissector frame 10 μm × 5 μm) was not observed in the look-up section, the synapse was counted. The number of synapses per unit volume (S*v*) was calculated using the following formula: S*v* = Total number of counted synapses/(Sum of Areas of disector frame) × Section thickness (Sterio, 1984). Since the density of neurons and synapses depended on the volume of the tissue, the number of synapses per neuron was estimated to correct the possible volume difference between groups. Using the Scion image software, the volume of molecular layer and granule cell layers were measured. The number of synapses per neuron was estimated using the following formula: Synapse/Neuron = (Synaptic density/Neuronal density) × (Volume of molecular layer/Volume of granule cell layer).

### 2.4 Statistical analysis

Statistical comparisons were performed using two-way ANOVA with post-hoc cell means comparisons for all experiments except for the paired-association test, which utilized nonparametric chi square analysis due to the binary nature of the performance index used. Multiple pair-wise comparisons were performed using Tukey’s HSD test. A probability value (*p*) less than 0.05 was considered statistically significant. The data are presented as mean ± SEM with individual data points also shown.

## 3 Results

### 3.1 EAS impaired on recognition memory in aged rats

Recognition of objects or people is an important cognitive ability in daily lives and has been recognized as a one of the major decline of cognitive aging (Robitsek et al., 2008; Burke et al., 2010; Arias-Cavieres et al., 2017). Recognition memory (RM) was tested using the discrimination index in the novel object recognition test. A two-way ANOVA revealed significant effect of stress in both the 6- and 72-h retention tests [Figures 2C,D; 6-h: *F*_(1,46)_ = 9.21, *p* = 0.004; 72-h: *F*_(1,46)_ = 5.77, *p* = 0.02] but no effect of aging in both 6-h and the 72-h retention test [Figures 2C,D; 6-h: *F*_(1,46)_ = 0.09, *p* = 0.75; 72-h: *F*_(1,46)_ = 3.06, *p* = 0.09]. There was a significant stress x aging interaction in both the 6- and the 72-h retention tests [6-h: *F*_(1,46)_ = 6.28, *p* = 0.02; 72-h: *F*_(1,46)_ = 4.31, *p* = 0.04]. Pairwise comparisons using Tukey HSD test revealed significant differences in SA vs. CA in the 6-h retention test (*p* = 0.005) and in SA vs. CA, SA vs. CY, and SA vs. SY in the 72-h retention test (*p*’s < 0.03) (Figure 2D).

Taken together, stress experienced during early adulthood produced an impairment of RM in aged rats, which was more evident in the long-term (72-h) retention of RM.

### 3.2 EAS impaired episodic-like memory in aged rats

To evaluate the episodic-like memory (EM), the rats were tested in the paired-association task, which requires discrimination between two temporally close events, pairs of flavor-location associations (Day et al., 2003). In a daily training session, the rats were briefly exposed to the cue flavor in the start box first, then entered the arena to find the corresponding location. All rats learned the procedural requirement to perform the task: the latency to arrive at the target location decreased throughout the sessions for all groups of rats [*F*_(5.29, 217.21)_ = 4.16, *p* < 0.05] (Supplementary Figure S1). At the end of the 10-day training, a probe test was conducted. The probe test was same as the training session except that the food reward was omitted from both well locations (Cued and Non-cued) and a new well location (Novel) was added as a distractor (Figure 3A). During the probe test session, there was no food in any of the wells to induce digging behavior solely based on the memory. Due to the individual variations in digging pattern (Figure 3B), we simplified the performance index by discriminating the success and failure of retrieving episodic-like memory based on the relative time allocation in the three wells. The retrieval was considered a “Success” if the rat allocated their digging time in the following order: Cued> Noncued> Novel and a “Fail” otherwise. Using this index, a nonparametric chi-square test of independence was performed to determine the relationship between stress and memory retrieval in each age group. The results show that there is a significant relationship between successful memory retrieval and the stress experience in aged [*χ*^2^(1, *N* = 17) = 5.61, *p < 0.05] but not in young rats [*χ*^2^(1, *N* = 30) =1.22, *p < 0.26], indicating that a significantly lower proportion of animals in the stress aged group performed the successful memory retrieval (Figure 3C). Taken together, these findings suggest that EAS effect on episodic-like memory, especially that requires precise discrimination among similar events, is more evident later in life, perhaps due to the combined effect of stress and aging.

### 3.3 Aging impaired trace fear conditioning but reversed in stress aged rats

Trace fear conditioning involves animals to associate an auditory conditioned stimulus (CS) and unconditioned stimulus (US) across a stimulus-free time gap. Multiple brain structures including the hippocampus have been indicated in trace fear memory formation (Seo et al., 2015; East et al., 2021; Trask et al., 2021). Trace fear memory is vulnerable to aging-related decline in humans and aged rats (McEchron et al., 2004; Moyer and Brown, 2006). A two-way ANOVA showed that during conditioning, there was no significant main effect of stress [*F*_(1,42)_ = 0.66, *p* = 0.42] nor aging [*F*_(1,42)_ = 0.37, *p* = 0.55] on freezing to the CS. However, the interaction between stress x aging was significant [Figure 4B; stress × aging: *F*_(1,42)_ = 19.21, *p* < 0.001]. Pairwise comparisons using Tukey HSD test revealed significant differences in SA vs. CA (*p* = 0.01), SA vs. SY (*p* = 0.003) (Figure 4A, right). SA group rats significantly froze more than CA and SY group.

After a 24-h period during the retrieval test, freezing levels were measured for 5 min in the same context as the conditioning but without the CS (context memory test), and for 7 min in the novel context with the 5 min CS (tone memory test). In the context test, a two-way ANOVA revealed that there was a significant main effect of aging [Figure 4C; *F*_(1,42)_ = 9.69, *p* = 0.003], but not stress [*F*_(1,42)_ = 1.76, *p* = 0.19] during context test. In addition, the interaction between stress x aging was significant [stress × aging: *F*_(1,42)_ = 4.04, *p* = 0.05]. Pairwise comparisons using Tukey HSD test revealed significant differences between CY and CA (*p* = 0.006). For the tone memory test, there was a significant main effect of aging [Figure 4D; *F*_(1,42)_ = 5.50, *p* = 0.02], but not stress [*F*_(1,42)_ = 0.02, *p* = 0.89] during tone test. The interaction between stress x aging was not significant [*F*_(1,42)_ = 2.07, *p* = 0.16]. In general, trace fear memory appears to be more susceptible to age-related alterations in the fear memory process within the brain.

### 3.4 Stress and aging impaired synaptic plasticity in DG

To investigate the neural underpinnings of memory dysfunction caused by stress and/or aging, we examined the synaptic plasticity by inducing Long-Term Potentiation (LTP) at the hippocampal PP-DG synapse. This synapse plays a crucial role in receiving major input information from the entorhinal cortex to the hippocampus. LTP was quantified by assessing the percentage changes from the baseline prior to high-frequency stimulation in the fEPSP slope (Figure 5A). The fEPSP slope was averaged for 80 min and expressed as a percentage of baseline measurement (Figures 5C,D). The statistical analysis, using a two-way ANOVA revealed a significant main effect of stress [Figure 5B; *F*_(1,22)_ = 6.98, *p* = 0.01] but not aging [*F*_(1,22)_ = 3.67, *p* = 0.07] on LTP. Additionally, there was a significant interaction between stress and aging [Figure 5B; stress × aging: *F*_(1,22)_ = 4.96, *p* = 0.04]. Pairwise comparisons using Tukey HSD test revealed that the fEPSP slopes of the SY, CA and SA animals were reduced compared to fEPSP slope of the CY group (SY, *p* = 0.003; CA, *p* = 0.03; SA, *p* = 0.01). These findings collectively signify that stress, aging, or their combined effect on LTP in the PP-DG synapse.

### 3.5 Neural density and synaptic density in DG reduced in the stress animals

To investigate potential neural structural alterations due to stress and/or aging, we evaluated neural density (Figure 6A) and synaptic density (Figure 6B) in a subset of animals (excluding those used in the LTP experiment to avoid the artificial effect of HFS) as described in the method section. A two-way ANOVA on the neural density revealed a significant main effect of stress [Figure 6A, bottom; *F*_(1,11)_ = 6.73, *p* = 0.02], indicating that stress had a significant impact on neural density. However, there was no significant main effect of aging [*F*_(1,11)_ = 1.35, *p* = 0.27]. Pairwise comparisons using Tukey HSD test revealed significant reduction in neural density for SY compared to CY (*p* = 0.015) reflecting the discernible influence of recent stress on the neural density of the dentate gyrus (Figure 6A, bottom).

To quantify synaptic density, the number of synapses per neuron was analyzed (Figure 6B). A two-way ANOVA revealed a significant main effect of aging [Figure 6B, bottom; *F*_(1,11)_ = 7.5, *p* = 0.02] and stress [*F*_(1,12)_ = 10.08, *p* = 0.009]. However, there was no significant interaction effect [*F*_(1,11)_ = 1.93 *p* = 0.19]. Pairwise comparisons using Tukey HSD test revealed significant differences revealed that the number of synapses per neuron in SA was significantly lower than all other groups [CA, SY, CY: *p*’s < 0.05, Figure 6B, bottom]. These results suggest that both stress and aging additively contribute to changes in synaptic density in stress aged animals.

## 4 Discussion

The results of the current study indicate that exposure to chronic mild stress during early adulthood contributes to the variability of cognitive aging. Stress aged rats exhibited impairments in recognition memory and episodic-like memory. Interestingly, trace fear memory, especially context memory, which is typically impaired by aging, was reversed by EAS in stress aged rats. Moreover, the reduction in synaptic density in the dentate gyrus middle molecular layer observed in the stress aged group may be relevant to imprecise memory discrimination.

Recognition memory is composed of two processes: recollection and familiarity. The recognition memory performance of aged individuals is variable, dependent on the recollection deficit and the compensatory increase in familiarity (Davidson and Glisky, 2002; Gallagher et al., 2006; Howard et al., 2006; Robitsek et al., 2008; Angel et al., 2016). In our study, object recognition memory remained intact in control aged rats, while EAS aged rats displayed significant impairment. This suggests that structural alteration induced by early adulthood stress and aging may disproportionately affect the recollection process compared to the familiarity process. The observed impaired precise memory retrieval in EAS aged rats may be related to a failure in pattern discrimination/separation (Wilson et al., 2006; Lupien et al., 2009; Yassa et al., 2010, 2011; Besnard and Sahay, 2016; Guo et al., 2018).

Episodic-like memory involves processing unique events and distinguishing similar events occurring at different times (Day et al., 2003; Bast et al., 2005). In EM performance, we observed a relatively small number of animals exhibiting a precise memory retrieval pattern (Cued > Noncued > Novel) in the stress aged group. Interestingly, stress young group showed a similar pattern but did not reach statistical significance. The prevalent imprecise memory performance in the stress aged group implies that this deficit may be related to the dysfunction of memory discrimination during neural information processing (Park et al., 2015; van Dijk and Fenton, 2018).

On the other hand, trace fear conditioning and context memory performance exhibited a different pattern compared to RM and EM. The effect of aging on context memory was significant only in control animals, consistent with previous studies (McEchron et al., 2004; Moyer and Brown, 2006; Dulka et al., 2020). Interestingly, the effect of aging on context memory was attenuated in stressed animals, perhaps due to the alterations in emotional processing in an array of extra-hippocampal circuits including the amygdala, medial prefrontal cortex, and entorhinal cortex (Runyan et al., 2004; Gilmartin and McEchron, 2005; Kochli et al., 2015; Song et al., 2015) or their interactions with the hippocampus. Importantly, the stress experiences enhance the emotional response or related behaviors (such as freezing) by strengthening the functional connectivity between the amygdala and the hippocampus (Rodrigues et al., 2009; Ghosh et al., 2013; Suvrathan et al., 2014). A similar, but not statistically significant pattern was observed in the tone memory test, indicating that emotional memory to the tone CS is less dependent on the hippocampal circuit than context memory performance (Rodrigues et al., 2009).

Previous research suggests a potential neurobiological mechanism underlying the observed impairment in RM and EM in EAS aged rats (Barnes, 1988; Geinisman et al., 1992; Kim and Diamond, 2002; Aleisa et al., 2006; Champagne et al., 2008; Arias-Cavieres et al., 2017; Dulka et al., 2020). These findings are consistent with the detrimental changes that occur in the hippocampal system due to stress and aging. Specifically, the dentate gyrus plays an essential role in the computational function of pattern separation for memory (Marr, 1971; Leutgeb et al., 2007; Neunuebel and Knierim, 2014; van Dijk and Fenton, 2018). The middle molecular layer of DG exhibits distinct changes influenced by experience and aging (Sharp et al., 1987; Andersen et al., 1996; Park et al., 2015). Synaptic plasticity, which is crucial for information processing in memory function, is susceptible to negative effects by stress and aging (Shors and Thompson, 1992; Shors et al., 1992; Baker and Kim, 2002; Kim and Diamond, 2002; Pavlides et al., 2002; Vouimba et al., 2004; Aleisa et al., 2006; Kosten et al., 2007). Impairment of long-term potentiation (LTP) in the dentate gyrus is a common observation in both stress and aging. Aged rats exhibit reduced input from cortical information processing (Sharp et al., 1987; Arias-Cavieres et al., 2017; Schreurs et al., 2017; Amani et al., 2021), and chronic stress in adults negatively affects LTP in the dentate gyrus (Kim and Diamond, 2002; Pavlides et al., 2002). Both stress and aging contribute to the impairment of LTP, which is consistent with previous studies. However, due to interaction of stress and aging, it is not possible to differentiate the effect of EAS on LTP from the combined effect or effect of aging. Further study is needed to explore the nuances of this relationship.

Investigating synaptic changes in the neural circuit related to memory dysfunction, our findings emphasize the specific impact of EAS on synapse density in the middle molecular layer of the DG. The reduced synaptic density per neuron provides evidence of malfunctioning DG synaptic capacity, revealing a difference between control aged and stress aged rats. The reduction in synaptic density in our results reflects both the number of synapses and a change in the volume of the molecular layer. These changes are in line with the previous studies that stress has effects on the atrophy of synapses and dendrites (Wang et al., 2012; Dayananda et al., 2023). Chronic mild stress exposure led to a decrease in the expression of synaptophysin in the dentate gyrus, indicating a detrimental effect on the integrity of the synapses (Wang et al., 2012). These morphological changes are associated not only with aging (Geinisman et al., 1992; Hara et al., 2012) but also with the enduring impact of remote life-long stress (Herrmann et al., 2012), and may be related to imprecise memory discrimination (Chenani et al., 2022; Wang et al., 2023).

**Figure 7:**
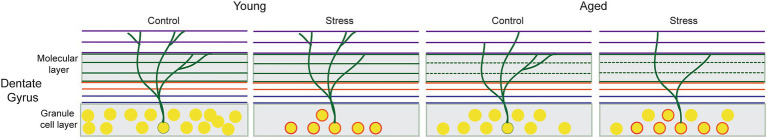
Hypothetical model of cellular alterations in the Dentate Gyrus following early adulthood stress (EAS) and aging. The reduced synaptic density represents combined effect of stress and aging, as symbolized by thinning of dendritic branches in the middle molecular layer. Changes in neural density might be less straightforward: the initial EAS induces not only a decrease in the number of cells but also cellular and molecular changes in survived neurons that persist throughout the entire adulthood (circled neurons). These changes might be responsible for suboptimal memory performance in stress aged animals that can be detected only with a sophisticated behavioral testing.

The impaired LTP that characterizes the synaptic plasticity of the DG implies that the neural circuits are less effective at processing new information. However, the cellular impairment due to stress in young animals was not clearly detected in all memory tests. Processing precise information required for memory discrimination among similar events may depend on a high level of synaptic capacity. In the current study, the result from synaptic density may represent the level of synaptic capacity. Overall, the pattern of behavioral impairment (combined effect of stress and aging on RM and aging effect in stressed animals on EM) coincides with the synaptic density impairment than neural density. To illustrate the difference between neural and synaptic density by which the pattern of behavioral impairments following stress and aging could be interpreted, we propose a model of neural and synaptic deterioration in Figure 7. In the figure, the reduced synaptic density in the middle perforant path represents the combined effect of stress and aging while the effect on neural density is more sophisticated: there might be a recovery of numbers after the initial EAS although the affected neurons remained suboptimal at the cellular and molecular level (circled neurons).

Three different hippocampus-dependent memory tasks revealed increased variability of cognitive function in EAS animals. Recognition memory, episodic-like memory, and trace-fear memory all involve the hippocampus, but are distinct in their substantial or partial dependence on the functional integrity of the hippocampus. Although the study primarily focused on the hippocampus dentate gyrus, it hinted at the potential involvement of compensatory circuits or alternative circuits adapting differentially to EAS. Further investigations are needed to address this aspect.

In conclusion, this current study demonstrates the enduring effects of early adulthood stress (EAS) on the cognitive aging process. The impact of EAS on recognition memory, episodic memory, and trace fear memory revealed a complex interaction with aging, increasing the variability of cognitive function. The unique contribution of our study lies in identifying specific synapse areas within the dentate gyrus associated with imprecise memory discrimination, providing valuable insight into the neural mechanisms involved. Consequently, our study provides an insight into how the course of cognitive aging in humans with various stress experiences could unfold over time. Stress experiences may interact with individual genetic differences, functioning either as a resilience factor or, at times, as exacerbating insults. Stress has the potential to increase inter- or intra-variability, impacting diverse brain structures. Coupled with the accumulating database regarding gene-behavior relations, the current study and subsequent studies employing a multitude of behavioral test batteries and circuit network analyses will contribute to delineating key factors influencing cognitive aging.

## Data availability statement

The raw data supporting the conclusions of this article will be made available by the authors, without undue reservation.

## Ethics statement

The animal study was approved by Animal Care and Use Committee/Korea University. This study was conducted in accordance with the local legislation and institutional requirements (Korea University).

## Author contributions

EunP: Conceptualization, Data curation, Formal analysis, Investigation, Writing – original draft, Writing – review & editing. YJ: Investigation, Writing – review & editing. EK: Investigation, Methodology, Writing – review & editing. EuiP: Investigation, Writing – review & editing. KL: Investigation, Writing – review & editing. IR: Supervision, Writing – review & editing. HK: Resources, Supervision, Writing – review & editing. J-SC: Conceptualization, Funding acquisition, Investigation, Resources, Supervision, Writing – review & editing.

## Footnotes

^1^ http://synapses.bu.edu/tools

## References

[ref1] Aleisa A. M. Alzoubi K. H. Gerges N. Z. Alkadhi K. A. (2006). Chronic psychosocial stress-induced impairment of hippocampal LTP: possible role of BDNF. Neurobiol. Dis. 22, 453–462. doi: 10.1016/j.nbd.2005.12.005, PMID: 16530419

[ref2] Alves N. D. Patricio P. Correia J. S. Mateus-Pinheiro A. Machado-Santos A. R. Loureiro-Campos E.. (2018). Chronic stress targets adult neurogenesis preferentially in the Suprapyramidal blade of the rat dorsal dentate gyrus. Brain Struct. Funct. 223, 415–428. doi: 10.1007/s00429-017-1490-3, PMID: 28852856

[ref3] Amani M. Lauterborn J. C. Le A. A. Cox B. M. Wang W. Quintanilla J.. (2021). Rapid aging in the Perforant path projections to the rodent dentate gyrus. J. Neurosci. 41, 2301–2312. doi: 10.1523/JNEUROSCI.2376-20.2021, PMID: 33514675 PMC8018768

[ref4] Andersen P. Moser E. Moser M. B. Trommald M. (1996). Cellular correlates to spatial learning in the rat Hippocampus. J. Physiol. Paris 90:349. doi: 10.1016/S0928-4257(97)87917-X, PMID: 9089511

[ref5] Angel L. Bastin C. Genon S. Salmon E. Fay S. Balteau E.. (2016). Neural correlates of successful memory retrieval in aging: do executive functioning and task difficulty matter? Brain Res. 1631, 53–71. doi: 10.1016/j.brainres.2015.10.009, PMID: 26541580

[ref6] Arias-Cavieres A. Adasme T. Sanchez G. Munoz P. Hidalgo C. (2017). Aging impairs hippocampal-dependent recognition memory and LTP and prevents the associated RYR up-regulation. Front. Aging Neurosci. 9:111. doi: 10.3389/fnagi.2017.00111, PMID: 28484388 PMC5402473

[ref9001] Baker K. B. Kim J. J. (2002). Effects of stress and hippocampal NMDA receptor antagonism on recognition memory in rats. Learn Mem, 9, 58–65. 11992016 10.1101/lm.46102 PMC155932

[ref7] Barnes C. A. (1987). Neurological and behavioral investigations of memory failure in aging animals. Int. J. Neurol. 21-22, 130–136. PMID: 2980684

[ref8] Barnes C. A. (1988). Aging and the physiology of spatial memory. Neurobiol. Aging 9, 563–568. doi: 10.1016/S0197-4580(88)80114-3 3062467

[ref9] Bast T. Da Silva B. M. Morris R. G. (2005). Distinct contributions of hippocampal NMDA and AMPA receptors to encoding and retrieval of one-trial place memory. J. Neurosci. 25, 5845–5856. doi: 10.1523/JNEUROSCI.0698-05.2005, PMID: 15976073 PMC6724786

[ref10] Besnard A. Sahay A. (2016). Adult hippocampal neurogenesis, fear generalization, and stress. Neuropsychopharmacology 41, 24–44. doi: 10.1038/npp.2015.167, PMID: 26068726 PMC4677119

[ref90002] Borcel E. Perez-Alvarez L. Herrero A. I. Brionne T. Varea E. Berezin V.. (2008). Chronic stress in adulthood followed by intermittent stress impairs spatial memory and the survival of newborn hippocampal cells in aging animals: prevention by FGL, a peptide mimetic of neural cell adhesion molecule. Behav Pharmacol, 19, 41–9. 18195593 10.1097/FBP.0b013e3282f3fca9

[ref11] Brunson K. L. Kramar E. Lin B. Chen Y. Colgin L. L. Yanagihara T. K.. (2005). Mechanisms of late-onset cognitive decline after early-life stress. J. Neurosci. 25, 9328–9338. doi: 10.1523/JNEUROSCI.2281-05.2005 16221841 PMC3100717

[ref12] Burke S. N. Ryan L. Barnes C. A. (2012). Characterizing cognitive aging of recognition memory and related processes in animal models and in humans. Front. Aging Neurosci. 4:15. doi: 10.3389/fnagi.2012.00015 22988437 PMC3439640

[ref13] Burke S. N. Wallace J. L. Nematollahi S. Uprety A. R. Barnes C. A. (2010). Pattern separation deficits may contribute to age-associated recognition impairments. Behav. Neurosci. 124, 559–573. doi: 10.1037/a0020893, PMID: 20939657 PMC3071152

[ref14] Champagne D. L. Bagot R. C. Van Hasselt F. Ramakers G. Meaney M. J. De Kloet E. R.. (2008). Maternal care and hippocampal plasticity: evidence for experience-dependent structural plasticity, altered synaptic functioning, and differential responsiveness to glucocorticoids and stress. J. Neurosci. 28, 6037–6045. doi: 10.1523/JNEUROSCI.0526-08.2008, PMID: 18524909 PMC6670331

[ref15] Chenani A. Weston G. Ulivi A. F. Castello-Waldow T. P. Huettl R. E. Chen A.. (2022). Repeated stress exposure leads to structural synaptic instability prior to disorganization of hippocampal coding and impairments in learning. Transl. Psychiatry 12:381. doi: 10.1038/s41398-022-02107-5, PMID: 36096987 PMC9468341

[ref16] Davidson P. S. Glisky E. L. (2002). Neuropsychological correlates of recollection and familiarity in Normal aging. Cogn. Affect. Behav. Neurosci. 2, 174–186. doi: 10.3758/CABN.2.2.174, PMID: 12455684

[ref18] Dayananda K. K. Ahmed S. Wang D. Polis B. Islam R. Kaffman A. (2023). Early life stress impairs synaptic pruning in the developing Hippocampus. Brain Behav. Immun. 107, 16–31. doi: 10.1016/j.bbi.2022.09.014, PMID: 36174883 PMC10497209

[ref17] Day M. Langston R. Morris R. G. (2003). Glutamate-receptor-mediated encoding and retrieval of paired-associate learning. Nature 424, 205–209. doi: 10.1038/nature01769, PMID: 12853960

[ref19] Drew L. J. Fusi S. Hen R. (2013). Adult neurogenesis in the mammalian Hippocampus: why the dentate gyrus? Learn. Mem. 20, 710–729. doi: 10.1101/lm.026542.112, PMID: 24255101 PMC3834622

[ref20] Dulka B. N. Pullins S. E. Cullen P. K. Moyer J. R. Jr. Helmstetter F. J. (2020). Age-related memory deficits are associated with changes in protein degradation in brain regions critical for trace fear conditioning. Neurobiol. Aging 91, 160–166. doi: 10.1016/j.neurobiolaging.2020.03.001, PMID: 32280031 PMC7232789

[ref21] Dvorak D. Chung A. Park E. H. Fenton A. A. (2021). Dentate spikes and external control of hippocampal function. Cell Rep. 36:109497. doi: 10.1016/j.celrep.2021.109497, PMID: 34348165 PMC8369486

[ref22] East B. S. Jr. Brady L. R. Quinn J. J. (2021). Differential effects of lateral and medial entorhinal cortex lesions on trace, delay and contextual fear memories. Brain Sci 12:34. doi: 10.3390/brainsci12010034 35053778 PMC8773659

[ref23] Engle J. R. Barnes C. A. (2012). Characterizing cognitive aging of associative memory in animal models. Front. Aging Neurosci. 4:10. doi: 10.3389/fnagi.2012.00010 22988435 PMC3439635

[ref24] Erickson C. A. Barnes C. A. (2003). The neurobiology of memory changes in Normal aging. Exp. Gerontol. 38, 61–69. doi: 10.1016/S0531-5565(02)00160-2 12543262

[ref25] Gallagher M. Colantuoni C. Eichenbaum H. Haberman R. P. Rapp P. R. Tanila H.. (2006). Individual differences in neurocognitive aging of the medial temporal lobe. Age (Dordr.) 28, 221–233. doi: 10.1007/s11357-006-9017-5 22253491 PMC3259151

[ref26] Geiller T. Royer S. Choi J. S. (2017). Segregated cell populations enable distinct parallel encoding within the radial Axis of the CA1 pyramidal layer. Exp Neurobiol 26, 1–10. doi: 10.5607/en.2017.26.1.1, PMID: 28243162 PMC5326710

[ref27] Geinisman Y. Detoledo-Morrell L. Morrell F. Persina I. S. Rossi M. (1992). Age-related loss of Axospinous synapses formed by two afferent systems in the rat dentate gyrus as revealed by the unbiased stereological dissector technique. Hippocampus 2, 437–444. doi: 10.1002/hipo.450020411, PMID: 1308200

[ref28] Geinisman Y. Ganeshina O. Yoshida R. Berry R. W. Disterhoft J. F. Gallagher M. (2004). Aging, spatial learning, and Total synapse number in the rat CA1 stratum Radiatum. Neurobiol. Aging 25, 407–416. doi: 10.1016/j.neurobiolaging.2003.12.001, PMID: 15123345

[ref29] Ghosh S. Laxmi T. R. Chattarji S. (2013). Functional connectivity from the amygdala to the Hippocampus grows stronger after stress. J. Neurosci. 33, 7234–7244. doi: 10.1523/JNEUROSCI.0638-13.2013, PMID: 23616532 PMC6619590

[ref30] Gillespie N. A. Hatton S. N. Hagler D. J. Jr. Dale A. M. Elman J. A. Mcevoy L. K.. (2022). The impact of genes and environment on brain ageing in males aged 51 to 72 years. Front. Aging Neurosci. 14:831002. doi: 10.3389/fnagi.2022.831002, PMID: 35493948 PMC9051484

[ref90001] Gilmartin M. R. Mcechron M. D. (2005). Single neurons in the medial prefrontal cortex of the rat exhibit tonic and phasic coding during trace fear conditioning. Behav Neurosci, 119, 1496–510. 16420154 10.1037/0735-7044.119.6.1496

[ref31] Gogtay N. Giedd J. N. Lusk L. Hayashi K. M. Greenstein D. Vaituzis A. C.. (2004). Dynamic mapping of human cortical development during childhood through early adulthood. Proc. Natl. Acad. Sci. USA 101, 8174–8179. doi: 10.1073/pnas.0402680101, PMID: 15148381 PMC419576

[ref32] Gray D. T. Barnes C. A. (2015). Distinguishing adaptive plasticity from vulnerability in the aging Hippocampus. Neuroscience 309, 17–28. doi: 10.1016/j.neuroscience.2015.08.001, PMID: 26255677 PMC4630086

[ref33] Gundersen H. J. Bagger P. Bendtsen T. F. Evans S. M. Korbo L. Marcussen N.. (1988). The new stereological tools: Disector, fractionator, Nucleator and point sampled intercepts and their use in pathological research and diagnosis. APMIS 96, 857–881. doi: 10.1111/j.1699-0463.1988.tb00954.x, PMID: 3056461

[ref34] Guo N. Soden M. E. Herber C. Kim M. T. Besnard A. Lin P.. (2018). Dentate granule cell recruitment of feedforward inhibition governs engram maintenance and remote memory generalization. Nat. Med. 24, 438–449. doi: 10.1038/nm.4491, PMID: 29529016 PMC5893385

[ref35] Hara Y. Park C. S. Janssen W. G. Roberts M. T. Morrison J. H. Rapp P. R. (2012). Synaptic correlates of memory and menopause in the hippocampal dentate gyrus in Rhesus monkeys. Neurobiol. Aging 33, E17–E28. doi: 10.1016/j.neurobiolaging.2010.09.014 PMC3031995 21030115

[ref36] Herrmann L. Ionescu I. A. Henes K. Golub Y. Wang N. X. Buell D. R.. (2012). Long-lasting hippocampal synaptic protein loss in a mouse model of posttraumatic stress disorder. PLoS One 7:E42603. doi: 10.1371/journal.pone.0042603, PMID: 22900032 PMC3416820

[ref37] Howard M. W. Bessette-Symons B. Zhang Y. Hoyer W. J. (2006). Aging selectively impairs recollection in recognition memory for pictures: evidence from modeling and receiver operating characteristic curves. Psychol. Aging 21, 96–106. doi: 10.1037/0882-7974.21.1.96, PMID: 16594795 PMC1749613

[ref38] Hultsch D. F. Strauss E. Hunter M. A. Macdonald S. W. S. (2008). Intraindividual variability, cognition, and aging. The handbook of aging and cognition, 3rd Edn. New York, NY: Psychology Press.

[ref39] Kim J. J. Diamond D. M. (2002). The stressed Hippocampus, synaptic plasticity and lost memories. Nat. Rev. Neurosci. 3, 453–462. doi: 10.1038/nrn849, PMID: 12042880

[ref9002] Kochli D. E. Thompson E. C. Fricke E. A. Postle A. F. Quinn J. J. (2015). The amygdala is critical for trace, delay, and contextual fear conditioning. Learn Mem, 22, 92–100. 25593295 10.1101/lm.034918.114 PMC4341367

[ref9003] Kosten T. A. Karanian D. A. Yeh J. Haile C. N. Kim J. J. Kehoe P.. (2007). Memory impairments and hippocampal modifications in adult rats with neonatal isolation stress experience. Neurobiol Learn Mem, 88, 167–76. 17543553 10.1016/j.nlm.2007.03.011

[ref9004] Kosten T. A. Lee H. J. Kim J. J. (2006). Early life stress impairs fear conditioning in adult male and female rats. Brain Res, 1087, 142–50. 16626646 10.1016/j.brainres.2006.03.009

[ref40] Leal S. L. Yassa M. A. (2015). Neurocognitive aging and the Hippocampus across species. Trends Neurosci. 38, 800–812. doi: 10.1016/j.tins.2015.10.003, PMID: 26607684 PMC5218997

[ref41] Leutgeb J. K. Leutgeb S. Moser M. B. Moser E. I. (2007). Pattern separation in the dentate gyrus and CA3 of the Hippocampus. Science 315, 961–966. doi: 10.1126/science.1135801 17303747

[ref42] Lupien S. J. Juster R. P. Raymond C. Marin M. F. (2018). The effects of chronic stress on the human brain: from neurotoxicity, to vulnerability, to opportunity. Front. Neuroendocrinol. 49, 91–105. doi: 10.1016/j.yfrne.2018.02.001, PMID: 29421159

[ref43] Lupien S. J. Mcewen B. S. Gunnar M. R. Heim C. (2009). Effects of stress throughout the lifespan on the brain, behaviour and cognition. Nat. Rev. Neurosci. 10, 434–445. doi: 10.1038/nrn2639 19401723

[ref44] Marks W. D. Yamamoto N. Kitamura T. (2021). Complementary roles of differential medial entorhinal cortex inputs to the Hippocampus for the formation and integration of temporal and contextual memory (systems neuroscience). Eur. J. Neurosci. 54, 6762–6779. doi: 10.1111/ejn.14737, PMID: 32277786 PMC8187108

[ref45] Marr D. (1971). Simple memory: a theory for Archicortex. Philos. Trans. R. Soc. Lond. Ser. B Biol. Sci. 262, 23–81. PMID: 4399412 10.1098/rstb.1971.0078

[ref46] Matud M. P. Diaz A. Bethencourt J. M. Ibanez I. (2020). Stress and psychological distress in emerging adulthood: a gender analysis. J. Clin. Med. 9:2859. doi: 10.3390/jcm9092859, PMID: 32899622 PMC7564698

[ref47] Mcechron M. D. Cheng A. Y. Gilmartin M. R. (2004). Trace fear conditioning is reduced in the aging rat. Neurobiol. Learn. Mem. 82, 71–76. doi: 10.1016/j.nlm.2004.06.002, PMID: 15341791

[ref48] McEwen B. S. (1999). Stress and hippocampal plasticity. Annu. Rev. Neurosci. 22, 105–122. doi: 10.1146/annurev.neuro.22.1.105 10202533

[ref49] McEwen B. S. (2011). Effects of stress on the developing brain. Cerebrum 2011:14. PMID: 23447783 PMC3574783

[ref50] Moyer J. R. Jr. Brown T. H. (2006). Impaired trace and contextual fear conditioning in aged rats. Behav. Neurosci. 120, 612–624. doi: 10.1037/0735-7044.120.3.612, PMID: 16768613

[ref51] Neunuebel J. P. Knierim J. J. (2014). CA3 retrieves coherent representations from degraded input: direct evidence for CA3 pattern completion and dentate gyrus pattern separation. Neuron 81, 416–427. doi: 10.1016/j.neuron.2013.11.017, PMID: 24462102 PMC3904133

[ref52] Papp M. Moryl E. Willner P. (1996). Pharmacological validation of the chronic mild stress model of depression. Eur. J. Pharmacol. 296, 129–136. doi: 10.1016/0014-2999(95)00697-4 8838448

[ref53] Park E. H. Burghardt N. S. Dvorak D. Hen R. Fenton A. A. (2015). Experience-dependent regulation of dentate gyrus excitability by adult-born granule cells. J. Neurosci. 35, 11656–11666. doi: 10.1523/JNEUROSCI.0885-15.2015, PMID: 26290242 PMC4540800

[ref54] Pavlides C. Nivon L. G. Mcewen B. S. (2002). Effects of chronic stress on hippocampal long-term potentiation. Hippocampus 12, 245–257. doi: 10.1002/hipo.1116 12000121

[ref55] Robitsek R. J. Fortin N. J. Koh M. T. Gallagher M. Eichenbaum H. (2008). Cognitive aging: a common decline of episodic recollection and spatial memory in rats. J. Neurosci. 28, 8945–8954. doi: 10.1523/JNEUROSCI.1893-08.2008, PMID: 18768688 PMC2585597

[ref56] Rodrigues S. M. Ledoux J. E. Sapolsky R. M. (2009). The influence of stress hormones on fear circuitry. Annu. Rev. Neurosci. 32, 289–313. doi: 10.1146/annurev.neuro.051508.135620, PMID: 19400714

[ref90005] Runyan J. D. Moore A. N. Dash P. K. (2004). A role for prefrontal cortex in memory storage for trace fear conditioning. J Neurosci, 24, 1288–95. 14960599 10.1523/JNEUROSCI.4880-03.2004 PMC6730343

[ref57] Sandi C. (2013). Stress and cognition. Wiley Interdiscip. Rev. Cogn. Sci. 4, 245–261. doi: 10.1002/wcs.1222, PMID: 26304203

[ref9007] Sandi C. Touyarot K. (2006). Mid-life stress and cognitive deficits during early aging in rats: individual differences and hippocampal correlates. Neurobiol Aging, 27, 128–40. 16298248 10.1016/j.neurobiolaging.2005.01.006

[ref58] Schreurs A. Sabanov V. Balschun D. (2017). Distinct properties of long-term potentiation in the dentate gyrus along the Dorsoventral Axis: influence of age and inhibition. Sci. Rep. 7:5157. doi: 10.1038/s41598-017-05358-1, PMID: 28698637 PMC5506024

[ref59] Segi-Nishida E. Suzuki K. (2022). Regulation of adult-born and mature neurons in stress response and antidepressant action in the dentate gyrus of the Hippocampus. Neurosci. Res. doi: 10.1016/j.neures.2022.08.010 36030966

[ref60] Seo D. O. Carillo M. A. Chih-Hsiung Lim S. Tanaka K. F. Drew M. R. (2015). Adult hippocampal neurogenesis modulates fear learning through associative and nonassociative mechanisms. J. Neurosci. 35, 11330–11345. doi: 10.1523/JNEUROSCI.0483-15.2015 26269640 PMC4532761

[ref61] Seo D. O. Pang M. H. Shin M. S. Kim H. T. Choi J. S. (2008). Hippocampal NMDA receptors are necessary for auditory trace fear conditioning measured with conditioned Hypoalgesia in rats. Behav. Brain Res. 192, 264–268. doi: 10.1016/j.bbr.2008.04.011, PMID: 18514922

[ref62] Sharp P. E. Barnes C. A. Mcnaughton B. L. (1987). Effects of aging on environmental modulation of hippocampal evoked responses. Behav. Neurosci. 101, 170–178. doi: 10.1037/0735-7044.101.2.170, PMID: 3580119

[ref90004] Shors T. J. Thompson R. F. (1992). Acute stress impairs (or induces) synaptic long-term potentiation (LTP) but does not affect paired-pulse facilitation in the stratum radiatum of rat hippocampus. Synapse, 11, 262–5. 1321993 10.1002/syn.890110311

[ref90003] Shors T. J. Weiss C. Thompson R. F. (1992). Stress-induced facilitation of classical conditioning. Science, 257, 537–9. 1636089 10.1126/science.1636089

[ref63] Smith T. D. Adams M. M. Gallagher M. Morrison J. H. Rapp P. R. (2000). Circuit-specific alterations in hippocampal Synaptophysin immunoreactivity predict spatial learning impairment in aged rats. J. Neurosci. 20, 6587–6593. doi: 10.1523/JNEUROSCI.20-17-06587.2000, PMID: 10964964 PMC6772954

[ref9005] Song C. Ehlers V. L. Moyer J. R. Jr. (2015). Trace Fear Conditioning Differentially Modulates Intrinsic Excitability of Medial Prefrontal Cortex-Basolateral Complex of Amygdala Projection Neurons in Infralimbic and Prelimbic Cortices. J Neurosci, 35, 13511–24. 26424895 10.1523/JNEUROSCI.2329-15.2015 PMC4588614

[ref64] Sterio D. C. (1984). The unbiased estimation of number and sizes of arbitrary particles using the Disector. J. Microsc. 134, 127–136. doi: 10.1111/j.1365-2818.1984.tb02501.x, PMID: 6737468

[ref65] Sun Y. X. Su Y. A. Wang Q. Zheng J. Y. Zhang C. C. Wang T.. (2023). The causal involvement of the BDNF-TRKB pathway in dentate gyrus in early-life stress-induced cognitive deficits in male mice. Transl. Psychiatry 13:173. doi: 10.1038/s41398-023-02476-5, PMID: 37225683 PMC10209152

[ref66] Suvrathan A. Bennur S. Ghosh S. Tomar A. Anilkumar S. Chattarji S. (2014). Stress enhances fear by forming new synapses with greater capacity for long-term potentiation in the amygdala. Philos. Trans. R. Soc. Lond. Ser. B Biol. Sci. 369:20130151. doi: 10.1098/rstb.2013.0151 24298153 PMC3843883

[ref67] Trask S. Ferrara N. C. Grisales K. Helmstetter F. J. (2021). Optogenetic inhibition of either the anterior or posterior retrosplenial cortex disrupts retrieval of a trace, but not delay, Fear Memory. Neurobiol Learn Mem 185:107530. doi: 10.1016/j.nlm.2021.107530, PMID: 34592468 PMC8595712

[ref68] Van Dijk M. T. Fenton A. A. (2018). On how the dentate gyrus contributes to memory discrimination. Neuron 98:E5. doi: 10.1016/j.neuron.2018.04.018 PMC6066591 29731252

[ref9006] Vouimba R. M. Yaniv D. Diamond D. Richter-levin G. (2004). Effects of inescapable stress on LTP in the amygdala versus the dentate gyrus of freely behaving rats. Eur J Neurosci, 19, 1887–94. 15078562 10.1111/j.1460-9568.2004.03294.x

[ref69] Wang H. S. Rosenbaum R. S. Baker S. Lauzon C. Batterink L. J. Kohler S. (2023). Dentate gyrus integrity is necessary for behavioral pattern separation but not statistical learning. J. Cogn. Neurosci. 35, 900–917. doi: 10.1162/jocn_a_01981, PMID: 36877071

[ref70] Wang S. Yuan Y. Xia W. Li F. Huang Y. Zhou Y.. (2012). Neuronal apoptosis and synaptic density in the dentate gyrus of ischemic Rats' response to chronic mild stress and the effects of notch signaling. PLoS One 7:E42828. doi: 10.1371/journal.pone.0042828 22912748 PMC3415399

[ref71] Willner P. Moreau J. L. Nielsen C. K. Papp M. Sluzewska A. (1996). Decreased hedonic responsiveness following chronic mild stress is not secondary to loss of body weight. Physiol. Behav. 60, 129–134. doi: 10.1016/0031-9384(95)02256-2 8804652

[ref72] Wilson I. A. Gallagher M. Eichenbaum H. Tanila H. (2006). Neurocognitive aging: prior memories hinder new hippocampal encoding. Trends Neurosci. 29, 662–670. doi: 10.1016/j.tins.2006.10.002, PMID: 17046075 PMC2614702

[ref73] Witter M. P. (2007). The Perforant path: projections from the entorhinal cortex to the dentate gyrus. Prog. Brain Res. 163, 43–61. doi: 10.1016/S0079-6123(07)63003-9 17765711

[ref74] Yassa M. A. Lacy J. W. Stark S. M. Albert M. S. Gallagher M. Stark C. E. (2011). Pattern separation deficits associated with increased hippocampal CA3 and dentate gyrus activity in nondemented older adults. Hippocampus 21, 968–979. doi: 10.1002/hipo.20808 20865732 PMC3010452

[ref75] Yassa M. A. Stark S. M. Bakker A. Albert M. S. Gallagher M. Stark C. E. (2010). High-resolution structural and functional MRI of hippocampal CA3 and dentate gyrus in patients with amnestic mild cognitive impairment. NeuroImage 51, 1242–1252. doi: 10.1016/j.neuroimage.2010.03.040, PMID: 20338246 PMC2909476

[ref76] Zhang K. Chen L. Li Y. Paez A. G. Miao X. Cao D.. (2023). Differential laminar activation dissociates encoding and retrieval in the human medial and lateral entorhinal cortex. J. Neurosci. 43, 2874–2884. doi: 10.1523/JNEUROSCI.1488-22.2023, PMID: 36948584 PMC10124959

