# Supplementary material for: Heterogenous effect of early adulthood stress on cognitive aging and synaptic function in the dentate gyrus
Source: Front Mol Neurosci. 2024 Apr 4;17:1344141. doi: 10.3389/fnmol.2024.1344141 (PMC11024304; doi:10.3389/fnmol.2024.1344141)
Supplement: Supplementary file 1 [file Data_Sheet_1.pdf]

frontiers

Supplementary Material

Heterogenous effect of early adulthood stress on cognitive aging and synaptic function in the dentate gyrus

Eun Hye Park[1,2], Yong Sang Jo[1], Eun Joo Kim[1,5], Eui Ho Park[3], Kea Joo Lee[4], Ji Woong Kim[3], Im Joo Rhyu[3], Hyun Taek Kim[1], June-Seek Choi[1]

* **Correspondence:** June-Seek Choi j-schoi@korea.ac.kr

1 Supplementary Data

Table 1. CMS schedule

<table>
<tr><th></th><th>SUN</th><th>MON</th><th>TUE</th><th>WED</th><th>THU</th><th>FRI</th><th>SAT</th></tr>
<tr><td>9 AM</td><td rowspan="6">Stroboscopic Lighting</td><td>Food Dep.</td><td rowspan="3">Food Deprivation</td><td>Light On</td><td rowspan="7">White Noise</td><td></td><td rowspan="3">Crowded Housing</td></tr>
<tr><td>10</td><td rowspan="23">Soiled Cage</td><td rowspan="3">Empty water bottle</td><td></td></tr>
<tr><td>11</td><td></td></tr>
<tr><td>12PM</td><td>Water Dep</td><td></td><td rowspan="7">Cage Tilt</td></tr>
<tr><td>13</td><td rowspan="6">Stroboscopic Lighting</td><td rowspan="8">Cage Tilt</td><td></td></tr>
<tr><td>14</td><td></td></tr>
<tr><td>15</td><td></td><td></td></tr>
<tr><td>16</td><td></td><td></td><td rowspan="17">Crowded Housing</td></tr>
<tr><td>17</td><td></td><td></td></tr>
<tr><td>18</td><td></td><td></td></tr>
<tr><td>19</td><td rowspan="4">White Noise</td><td></td><td rowspan="7">Food & Water Deprivation</td><td rowspan="5">White Noise</td></tr>
<tr><td>20</td><td></td></tr>
<tr><td>21</td><td></td><td rowspan="12">Light Off</td></tr>
<tr><td>22</td><td rowspan="11">Food Deprivation</td></tr>
<tr><td>23</td><td></td></tr>
<tr><td>0 AM</td><td></td><td></td></tr>
<tr><td>1</td><td></td><td></td></tr>
<tr><td>2</td><td></td><td rowspan="3">Stroboscopic Lighting</td><td rowspan="5">White Noise</td></tr>
<tr><td>3</td><td rowspan="3">White Noise</td></tr>
<tr><td>4</td></tr>
<tr><td>5</td><td rowspan="4"></td></tr>
<tr><td>6</td><td></td></tr>
<tr><td>7</td><td></td><td></td></tr>
<tr><td>8</td><td></td><td></td></tr>
</table>

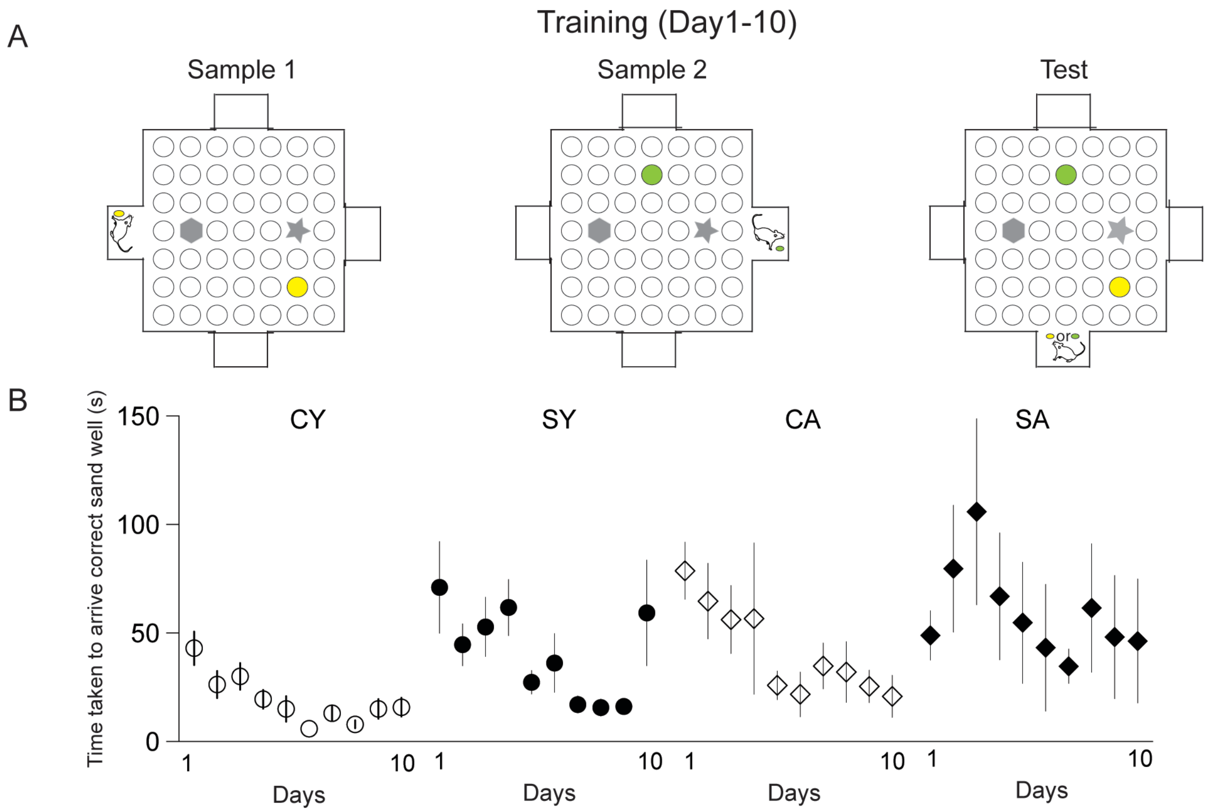

S1) Episodic-like memory test using event arena. A) Daily training session consisted of two sample trials and one test trial. Two gray marked locations landmarked as a spatial cue. Yellow marked sand well opened at first sample trial. Green marked well opened with different food flavor at the second sample trial. Time taken to arrive at the correct sand well containing food pellet was measured (Figure 3A). All groups showed the reduced time to arrive at the correct sand well over training days (Trials: $F_{(5.29,\ 217.21)} = 4.16$, $p = 0.001$). Significant main effect of aging ($F_{(1,\ 41)} = 4.97$, $p = 0.03$).

Table 2. Event arena training schedule (location and flavor)

Day	Flavor	Location
Training day1	Orange, Coconut	(5,2) (3,3)
Training day2	Vanilla, Bacon	(6,6) (3,4)
Training day3	Berry, Marshmallow	(2,6) (2,2)
Training day4	Almond, Kiwi	(2,1) (6,1)

Training day5	Pinacolada, Chocolate	(4,3) (2,6)
Training day6	Banana, Apple	(5,5) (2,3)
Training day7	Pear, Strawberry	(1,2) (1,5)
Training day8	Grape, Lemon	(5,4) (7,5)
Training day9	Ginger, Banana+chocolate	(5,1) (7,3)
Training day10	Cherry, Apple+chocolate	(3,5) (7,4)
Probe test	Melon, Mango+tropical punch	(3,2) (6,3)
